# Effect of herbal extracts in animal nutrition as feed additives

**DOI:** 10.1016/j.heliyon.2024.e24973

**Published:** 2024-01-26

**Authors:** Wondimagegn Tadesse Alem

**Affiliations:** Department of Animal Science, Kebri Dehar University, Ethiopia

**Keywords:** Feed additives, Herbal extract, Nutrition, Performance, Productivity

## Abstract

This paper is reviewed with the objective to understand the effect of herbal extracts on animal performance as feed additives. The number of both external and internal factors which affects the production and productivity of animals obviously includes nutrition as a major factor. Feed additives are products used in animal nutrition to increase the quality of feed and animal-derived foods, as well as the performance and the health of animals. Plant extracts as feed additives are described as herbal-derived components added to ration to improve livestock performance and product quality. Many herbal extracts contain chemical components that have antioxidant, antimicrobial, anti-inflammatory, anticoccidial and anthelmintic properties to improve ruminal microbial activity, diet palatability and stimulate digestion. Bioactive chemicals found in nature, such as flavonoids and glucosinolates isoprene derivatives, are primarily responsible for the qualities of plant extracts. Plant extracts are commonly added to feed to increase palatability, productivity and to inhibit lipid oxidation. When added to meals, herbal extracts aid to decrease rancidity, delay the generation of hazardous oxidation products, and retain nutritional quality. It is concluded that; herbal extracts are important to improve growth performance and product quality.

## List of Abbreviation

DNA: Deoxyribonucleic acid
GI: Gastro-Intestine
GIT: Gastro Intestinal Tract
RNA: Ribonucleic acid

## 1 Introduction

Farm animals farming is encouraged by way of a selection of factors; include negative nutrition, high prevalence of illnesses, terrible genetic resource improvement and management and negative market infrastructure. From the ones inadequate substances of feeds for the existing livestock population, negative fine of the available feed resources, seasonal variant in availability of feeds and water shortages are the main elements that make contributions to the low production and productivity of cattle inside the Ethiopia [1].

Feed additives are merchandise introduced to feed to enhance feed first-rate and animal-derived ingredients, in addition to the overall performance and fitness of the animals. There are numerous plant herbal extracts as feed components were explored by way of scientists to enhance animal fitness and production which include cinnamon, garlic, ginger, yucca, turmeric and others herbs [2]. pronounced that administration of plant natural extracts in animal food regimen undoubtedly influenced animal performances, carcass traits, biochemical and immune reaction of animals. Herbs consisting of cinnamon, peppermint, cloves nutmeg additionally include antioxidant residences (polyphenols, flavonoids, and terpenoids) functioned to prevent oxidative pressure and diseases [3].

Phytogenic feed additives (PFA) are commonly defined as numerous plant secondary compounds (%) and metabolites with beneficial consequences on animal fitness and production, consisting of feed and animal merchandise [4]. The amount of energetic compounds in plant-derived feed components would possibly vary significantly based on plant portion employed, harvesting season, and geographical beginning. The benefits of natural extracts for animals consist of elevated digestive secretions, boom nutrient digestibility and absorption, enhance GI (gastrointestinal) microorganisms, stimulation of immune device, antibacterial sports, coccidiostatic, anthelmintic, antiviral, anti-inflammatory, and antioxidant residences [5]. As a result, the purpose of this studies is to study the outcomes of herbal extracts as feed components on animals feed.

## 2 Literature review

### 2.1 Plant extracts, their properties and active compounds

Plant extracts are plant-derived substances added to feed to improve increase overall performance and product excellent. Extracts, spices, vital oils and oleoresins are categorised in step with their origins and procedures (Table 1). Plant extracts include a selection of materials, and the energetic secondary plant metabolites are normally isoprene derivatives and flavonoids [6]. The type and level of various biological activities exhibited via any plant extract, however, is dependent on a ramification of things such as geographical place, time of collection, soil situations, harvesting time, moisture content material, drying method, storage conditions, and publish-harvest method [7]. The quantity of active compounds in herbal extracts varies extensively relying on the natural portion, geographic origin, plant maturity stage, preservation methods and length, garage and extraction methods [8].

**Table 1:** The active components and functions of several herbal feed additives.

Active compounds	Herbal plant	Utilized parts	Function
Eugenol	Clove (*Syzygium aromaticum*)	Flower	Appetizer, digestion stimulant, antiseptic
Cinnamalde-hyde	Cinnamon (*Cinnamomum zeylanicum*)	Leaf	Appetizer, digestive stimulant, antiseptic
Linalol	Coriander (*Coriandrum sativum*)	Leaf-seed	Appetite enhancer, digestive stimulant
Anethol	Anise (*Pimpinella anisum*)	Fruit	Digestive stimulant, galactagogue
phtalides	celery (*Apium graveolens*)	Fruit, leaf	Appetizer, digestive stimulant
Capsaicin	Capsicum **(***Capsicum annuum*)	Fruit	Reduce pain and swelling
zingerone	Ginger (*Zingiber officinale*)	rhizome	Appetizer, Anti-oxidant, stress reducer
Allicin	Garlic (*Allium sativum*)	Buld	Enhance gut activity, promote growth in livestock and poultry.
Cineol	Rosemary *(Rosmarinus Officinalis)*	Leaf	Anti-bacterial and anti-microbial, neurological protection
Thymol		Whole plant	Pharmaceutical application and exhibit antimicrobial, antioxidant, anti-carcinogenesis, anti-inflammatory and immune-stimulants

### 2.2 Plant extracts as feed components in animal vitamins

Feed additive supplements, along with nutrients, play a strategic position in farm animals control. In latest decades, a growing interest in natural plant feed additives (PFA) is riding studies towards plant extracts, crucial oils (EO), and by means of-products of plant foundation as options to synthetic vitamins in cattle nutrients for his or her capability positive consequences on animal health and productivity [10]. Secondary metabolites discovered in plant extracts including tannins, saponins, flavonoids and important oils have the potential to alter digestive tract metabolism and enhance animal production performance [11]. [12] investigated numerous nearby potential herbs in Aceh province inclusive of Vernonia amygdalina Del., Calotropis gigantean, Syzygium oleana, Syzygium cumini, L and concluded that each one phytogenic may be used as feed additives because of its antimicrobial and antioxidant sports.

### 2.3 Outcomes of herbal extracts on animals

#### 2.3.1 Antimicrobial effect

Animals are much less uncovered to toxins of microbiological starting place due to increased digestive system health. Rumen microbial hobby has shown that using plant extracts and secondary plant metabolities which may offer an opportunity to ruminal modifiers for his or her capacity to enhance the use of energy and protein within the rumen [13]. Secondary metabolites Diagnosed in medicinal vegetation may have antibacterial homes that help in resistance prevention [14].

Herbs such as Allium sativum, Angelica dahurica, Anguisorba officinalls, Artemisia argyi, Coptis chinensis, Dictamnus dasycarpus, Fraxinus rhynchophylla, Geranium thunbergii, Hydrastis canadensis, Phellodenron amurense, Polygonum cuspidatum, Scutellria baicalensis and Sophora flavesens include important flavonoid additives, baicalein, limonene, cinnamaldehyde, carvacrol or eugenol which exerts antimicrobial effect together with different supportive herbs have antibacterial against Salmonella spp. And E. Coli, as well as gram-superb micro organism such as Staphylococcus spp. And Streptococcus spp. Antibacterial, anti-inflammatory, anti-oxidative, and anti-parasitic effects of phytobiotics were investigated [15].

The ethanol extracts of Withania somnifera L., Ageratum conyzoides L., and Becium obovatum (E. Mey. Ex Benth. In E. Mey) N.E. Br leaves, in addition to Pentas lanceolata root, have antibacterial action against Grampositive bacteria, S. Aureus, however no longer E. Coli. As example Pentas lanceolata ethanol extracts had the largest boom inhibition quarter in opposition to Gram-fantastic micro organism, S. Aureus, with inhibition zones of 15.67 and 18.67 mm at concentrations of fifty mg/mL and one hundred mg/mL, respectively [7]. Citrus sinensis (orange) peel extracts have remarkable antibacterial pastime against gram positive and gram negative micro-organism, in addition to antifungal activity against Candida albicans [16].

#### 2.3.2 Antioxidant impact

Natural extracts revealed an antioxidant impact by means of elevated the expression of the antioxidant protein heme oxygenase-1 (HO-1) (Table 2), superoxidase dismutase (SOD) and catalase (CAT) [17]. Antioxidants are materials that put off and inhibit lipid oxidation, and while added to food, they useful resource to lessen rancidity, prevent unsafe oxidation merchandise from forming, and preserve nutritional quality [18]. Flavonoids and phenolic compounds in plants own more than one organic results consisting of antioxidant, anti inflammatory, antimicrobial and antiallergic sports [19]. Phytogenic feed additives comprising unmarried or combinations of components provide promising feed additives with mentioned anitioxidant capacity [20].

**Table 2:** Anti-oxidant action of phytogenic plants in Animals.

Phytogenic plants	Anti-oxidant activities	Source
Toona sinensis	Diphenyl-1-picrylhydrazyl scavenging activity, inhibition of lipid peroxidation activity	[24]
Echinacea purpurea	Diphenyl-1-picrylhydrazyl scavenging activity	[25]

Consistent with [21], consuming natural ingredients boosts serum antioxidant enzyme sports like glutathione peroxidase tiers. The addition of rosemary to layer diets seems to have boosted the antioxidant capacity of the birds [21]. Rosemary carries a ramification of useful phenolic compounds, inclusive of carnosol, carnosic, and rosmarinic acids, in addition to other compounds with antioxidant, anti-cancer, and anti inflammatory homes [22]. Subcutaneous injection with 25 mg/kg body weight of saffron petal extract significantly reduces ldl cholesterol degree within the lamb plasma compared with those fed basal diet on my own [23].

#### 2.3.3 Anti-inflammatory impact

Plant extracts were used as herbal therapeutic agents against irritation, characterized via an overproduction of inflammatory mediators consisting of ROS and seasoned-inflammatory cytokines [17]. Curcuma, crimson pepper, black pepper, cumin, cloves, nutmeg, cinnamon, mint, and ginger extracts were determined to have anti inflammatory residences. Phenols, terpenoids, and flavonoids are the principle anti-inflammatory lively chemical substances. Aloe Vera has antibacterial and antiprotozoal homes that exceptionally help in controlling the diseases coccidosis in fowl [26]. The plant incorporates flavonoid and genins which have anti-edematous impact in the acute segment of irritation [27]. Graded dose 2 hundred and 400 mg/kg confirmed a notably vast reduction within the weight of granuloma in assessment with manipulate organization [27]. The observe on Antinociceptive and anti-inflammatory outcomes of Urtica dioica indicates that, the U. Dioica leaf extract at doses of 100, 200 and four hundred mg/kg inhibited abdominal twitches by 41 %, sixty four% and 81 %, respectively [28].

#### 2.3.4 Herbal feed additives as immune-stimulant

Immune-modulating natural plants are used to provide an alternative to standard chemotherapy for a selection of disorders, in particular the ones involving immunodeficiency [29,30]. Flora such as echinacea, licorice, garlic, and cat's claw include chemical substances that stimulate the immune gadget. Those herbs can boost lymphocyte, macrophage, as well as sell phagocytosis and result in interpheron production. Tocopherols, lutein, beta-carotene, and retinol concentrations in plasma and tissue of broilers were raised by way of oxidized fat. Critical oils derived from medicinal flowers boost the immune gadget and can regulate the mucosa of the duodenum, ensuing in tremendous effects on animal [31]. In keeping with [32], the Nile tilapia fish treated with turmeric, rosemary and thyme plant extracts elevated in haematocrit, leukocrit ranges and nitrobule tetrazolium hobby (NBT) than control group. Medicinal herbs may be effective on immunestimulators.

#### 2.3.5 Effect on feed palatability, digestion and productivity of non-ruminant animals

Herbal extracts have been validated to provide considerable advantages in the digestive tracts, which include laxative consequences and flatulence prevention, stimulation of digestive secretions, and accelerated enzyme interest [33]. [34] stated that feed intake is not best prompted by the palatability of the feed but additionally prompted via the smell, test, texture, colour of feed and balance of the dietary content material. Capsaicin, has been determined to boom pancreatic and intestinal enzyme output in non-ruminants via selling salivation (producing amylase) [5]. Cinnamaldehyde, had a stimulating impact on pancreatic enzymes and prolonged feed retention time in the stomach in pigs because it promoted decrease gastric motility. As an end result, higher digestive enzyme pastime supported stepped forward nutrient digestibility and availability [5].

Flavonoids, phenols, glycosides, coumarins, saponins, terpenes, alkaloids, and anthracene, amongst different biologically active compounds found in herbs, have a multidirectional have an effect on at the body. Herbs could make feed extra appealing to devour [35]. Evidence has additionally showed that plant-primarily based merchandise can manipulate E. Coli populace inside the ileum of pigs and L. Intracellularis in pig feces [36]. Whilst utilized in excessive concentrations, vital oils may have sturdy scents or ugly aromas. The addition of herbals to pig feed has the immediate impact of stimulating appetite. Currently, it changed into determined that menthol (Mentha arvensis) stronger perfect protein and amino acid digestibility, thus feed efficiency, in weaned piglets [37], and black paper improved broiler chicken performance [38].

[39] tested that, Aloe vera herbal merchandise will increase the digestion methods, help to combat towards bacteria and virus and will increase the antibody titers towards Newcastle and coccidiosis sickness in broiler chickens. Natural bioactive combination composied of lemon, onion and garlic juice progressed trendy health, egg weight, number of egg/bird, feed conversion efficiency and decrease the blood cholesterol of laying hens [40]. Feed conversion efficiency on layers is expanded with the aid of increasing stage of garlic powder supplementation in diets [41]. Recently, it become discovered that administration of red ginger and brotowali extracts separately and blended both extracts in consuming water resulted in excellent frame weight benefit, feed conversion ratio, carcass percentage, belly fats percent and cooking loss percentage of broilers [42] (Table 3).

**Table 3:** Effect of different herbal extracts on poultry.

Herbal extracts	Level	Effect on poultry	Source
Amla fruit powder		Decreased *E. coil* bacteria and increase *Lactobacilli* microbial load.	[43]
	0.25, 0.5, 0.75 and 1 % of diet	Lowest serum cholesterol value in broilers, hetrophils count reduced.	
Moringa oleifera leaf meal	0.5kg/100 kg diet	Improvement in egg production and feed conversion ratio.	[44]
Aloe vera poplysaccharides	100, 200, 300 mg/kg BW	Daily weight gain, anti-coccidial indices is higher.	[45]
Olive leaf powder	1, 2, or 3 %	Increased final body weight of hens, increase yellowness in yolk colur and decrease yolk cholesterol level.	[46]
Bioactive mixture of lemon, onion and garlic juice	1, 2,3 %	Improve feed conversion efficiency, number of egg/hen, percentage of egg production, egg weight and yolk color.	[40]
Red ginger, brotowali extract	1 % ginger, 5.12 g/kg BW brotowali and both in drinking water	Administration of both extract resulted in BW (1100.28g/head), feed conversion ratio (1.37), carcass percentage (83.58 %), abdominal fat (0.56 %), and cooking loss (8.56 %).	[42]

#### 2.3.6 Effect on rumen microbial activities

Rumen is an essential digestive organ in farm animals, which houses a complicated environment composed of a massive wide variety and a variety of microbial species, commonly along with bacteria, protozoa and fungi [47]. [48] mentioned that plant extracts are capability rumen modifiers because of their antimicrobial hobby against microorganisms. The huge software of plant extracts for decreasing ruminal methane emissions supports their position as rumen modifiers [49]. Plant extracts like garlic powder at dose rate of 250 and 300 mg/kg frame weight enhance basic feed intake, frame weight advantage, common every day advantage, body circumstance rating and feed conversion efficiency in buffalo calves [50] (desk 4). Supplementing feed with medicinal and aromatic natural plants caraway (Carum carvi), fennel (Foeniculum vulgare) and Melissa (Melissa Officinali) significantly growth IVDMD, IVOMD, metabolic electricity, overall gas, unstable fatty acid and rumen fermentation especial at stage of 7 % than control organization [13] (Table 4).

**Table 4:** Result review on effect of herbal extract on ruminant animals.

Authors	Used herbal extracts	Outcome with respect in rumen animals
[51]	Rosemary extract at 28 g	Significant increase in milk and milk lactose yield, rumen fermentation, superoxide dismutase concentration and decrease somatic cell count and malonaldehyde in high milk producing dairy cows.
[55])	Olive leaf extract	Rumen ciliate protozoans quantity decreased, number of bacteria and starch degradation increased. Both gram + ve and gram -ve are inhibited by olive leaf (Olea europea) extract.
[50]	Garlic supplementation with level of 250, 300 mg/kg BW.	Enhance overall feed intake, body weight gain, average daily gain, body condition score and feed conversion efficiency in buffalo calves.
[13]	caraway (*Carum carvi*), fennel (*Foeniculum vulgare*) and Melissa (*Melissa Officinali*) at level 1,3,5,7 %	Gas production per DM, short chain fatty acid, metabolizable energy, volatile fatty acid, In vitro digestibility of ruminant animal was significantly increased with level of extracts.
[56]	Essential oil on methane production and diversity of rumen microbial population.	Methane and other gas production is reduced as the dose of essential oil is increased. Essential oils altered the composition of archaeal and bacterial populations to varying degrees, according to denaturing gradient gel electrophoresis analyses.
[57]	Flavonoids on rumen fermentation activity, methane production and microbial population.	Total protozoa and methanogen populations were dramatically reduced by flavonoids. Flavones, myricetin, catechin, rutin, and kaemferol drastically lower the population of practically every rumen microbe.
[54]	Essential oil on rumen fermentation and protozoal counts.	In vitro tests of essential oils revealed antibacterial properties and lowered protozoal numbers. *Zingiber officinale had greater protozoal counts, but other essential oils had lower protozoal counts.*

Rosemary extract supplementation advanced milk yield and milk lactose yield, probable by using enhancing propionate production within the rumen, improving the antioxidant popularity in excessive-producing dairy cows, decreased the relative abundance of Prevotella and extended the abundance of numerous genera associated with nutrient degradation and fermentation [51] (Table 4). Fermentation product (VFA) extensively accelerated with supplementation of low degrees of tea saponin (10 g/livestock/day). These changes are in all likelihood attributable to changes in the relative abundance of microbial species involved in carbohydrate decomposition, found inside the LT remedy institution, along with Actinobacteria, Saccharomyces and Aspergillus [52]. Saponin supplementation in sheep also growth digestibility of organic remember, impartial detergent fibre and acid detergent fibre through nine.6 %, 27. Nine% and 38 %, respectively [53]. Because of their selective effect on precise rumen microorganisms, specially micro organism, important oils have a reduction in protein and starch degradation in addition to a suppression of amino acid degradation in the rumen (Table 4) [54].

## 3 Conclusion and future perspective

Feed additives are substances which might be delivered to animal feed to improve the exceptional of the feed, the best of animal-derived meals, or the performance and health of the animals. Plant extracts are one of the feed components utilized in farm animals feed. They may be crucial to improve growth performance and product great because of they use as antioxidant, antimicrobial, anti-inflammatory, anti-coccidial, anthelmintic residences in addition to enhancing ruminal microbial hobby, food plan palatability and stimulate digestion. Bioactive chemical substances determined in nature, consisting of flavonoids and glucosinolates isoprene derivatives, are frequently chargeable for the traits of plant extracts. The quantity of lively compounds in plant extracts varies notably due to natural component, geographic origin, herbal maturity level, maintenance strategies and length, garage and extraction processes.

Different extracts has different importance in animals. Therefore; it is recommended that.

• The producer should consider the extraction method, sources of extract, part of extract, etc, because composition and effectiveness is affected by those factors.
• The provider should consider the taste preference of different species of animals when using as appetite stimulant since different herbs and spices affect digestion differently.
• We have to consider the type or part and concentration of extract, type of animals and purposes to be provided during supplementation because the different extracts affect the animal production in different way.
• Excessive supplementation of herbal extracts should be avoided, because they can have negative effect.

## Ethics approval and consent to participate

Not applicable.

## Funding

No external funding.

## Consent for publication

Not applicable.

## Author information

Department of Animal Science, College of Dry-Land Agriculture, Kebri Dehar University, Ethiopia.

## CRediT authorship contribution statement

**Wondimagegn Tadesse Alem:** Writing – review & editing, Writing – original draft, Visualization, Validation, Supervision, Software, Resources, Project administration, Methodology, Investigation, Funding acquisition, Formal analysis, Data curation, Conceptualization.

## Declaration of competing interest

The authors declare that they have no known competing financial interests or personal relationships that could have appeared to influence the work reported in this paper.
